# Differential Diagnosis of Rare Diseases Involving Bilateral Lower Extremities with Similar ^99m^Tc-MDP Bone Scan Patterns: Analysis of a Case Series

**DOI:** 10.3390/diagnostics12040910

**Published:** 2022-04-06

**Authors:** Zhenkui Sun, Chentian Shen

**Affiliations:** Department of Nuclear Medicine, Shanghai Jiao Tong University Affiliated Sixth People’s Hospital, Shanghai 200233, China; sun77126@163.com

**Keywords:** bone scan, bilateral lower extremities, imaging characteristics, differential diagnosis

## Abstract

Here, we reported a panel of rare diseases involving bilateral lower extremities with similar imaging patterns on ${}^{99m}$Tc-MDP bone scans. Glucose-6-phosphate dehydrogenase deficiency (G-6PD deficiency), Gaucher disease (GD), steroid-induced osteonecrosis, progressive diaphyseal dysplasia (PDD), Erdheim–Chester disease (ECD) and Langerhans cell sarcoma (LCS) were included and imaging characteristics were analyzed. The rare properties of these diseases and mimicking features on ${}^{99m}$Tc-MDP bone scans rendered differential diagnosis difficult but necessary. We believe that the rarely known imaging features of the reported diseases will undoubtedly help nuclear medicine physicians make differential diagnoses in clinical practice.

## 1. Introduction

Although frequent deformities are encountered in pediatric orthopedic departments, diseases involving bilateral lower extremities are relatively rare in clinical practice. Clinical and radiological phenotypic characterizations are the baseline tools of diagnosis. However, the expanding availability of bone scintigraphy may help the physicians to better understand these diseases. In diseases such as hyperparathyroidism, typical imaging characteristics with hypercalcemia and hypophosphatemia usually enable a definitive diagnosis. Hypertrophic pulmonary osteoarthropathy (HPO) can likely be diagnosed based on clinical symptoms (unremitting pain, edema, and erythema in the extremities) and radiographic findings (radiography reveals periosteal membrane thickening and periosteal new bone formation, and bone scintigraphy shows bracelet-like or diffusely increased uptake throughout most of the skeleton). However, the imaging features of many other diseases may be disappointingly nonspecific, and the possible diagnostic considerations initially may seem extensive. In such cases, the use of a systematic approach can help narrow down the differential diagnosis.

Diseases involving bilateral lower extremities may be classified in one of the following general diagnostic categories: metabolic bone disorders, haematological systemic disorders, genetic developmental diseases, tumors, trauma, autoimmune diseases, and inflammatory lesions. Although these categories comprise a plethora of possible diagnoses, an orderly list of differential considerations may be constructed by considering the patient’s age, laboratory examination, salient imaging features, and clinical manifestations.

The current article is based on our experience with diseases involving bilateral lower extremities. It is not intended as a comprehensive review, but rather as an overview, with emphases on lesions that are more common or relatively more common and on diagnoses that may be suggested by bone scintigraphy and radiologic imaging.

## 2. Materials and Methods

Ethical review and approval were waived for this study due to its retrospective nature. Informed consent has been waived for most patients except for those whose medical images were used in the current study. Bone SPECT was acquired 3–3.5 h after intravenous administration of radiotracer technetium ${}^{99m}$Tc methylene diphosphonate (${}^{99m}$Tc-MDP) (7.4 MBq/kg). All hybrid SPECT/CT examinations were obtained on a GE Discovery NM/CT 670 (GE Healthcare, GE Medical Systems Israel, Hafa, Israel) scanner. These hybrid SPECT/CT images integrated the functional images of SPECT with precise anatomical detail of multi-slice high-resolution CT. Imaging data were reconstructed by incorporating two powerful software packages: Volumetrix 3D and Volumetrix IR. MRI examinations were obtained on a 3T scanner (Achieva 3.0T; Philips Healthcare, Best, The Netherlands). ${}^{18}$F-FDG PET/CT scanning was performed after an intravenous injection of 3–4 MBq/kg ${}^{18}$F-FDG, followed by a one hour uptake phase. No intravenous contrast agent was administered. ${}^{18}$F-FDG PET/CT imaging was performed using a dedicated GE Discovery PET/CT scanner including 64 slice CT scanners with a dedicated PET (BGO plus crystal).

## 3. Results and Discussion

### 3.1. G6PD Deficiency

G-6PD deficiency is one of the most common human enzyme defects that causes many biochemical and clinical phenotypes, including neonatal jaundice, chronic haemolysis and acute haemolytic anaemia [1]. However, ${}^{99m}$Tc-MDP bone scan and radiological patterns of G-6PD deficiency have rarely been reported [2]. Therefore, we first reported a G-6PD deficiency case that was previously misdiagnosed as fibrous dysplasia.

**Figure 1:**
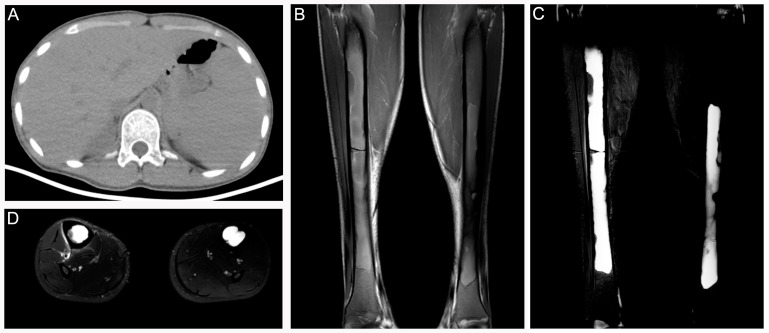
Patient 1: (**A**) Axial CT demonstrated marked hepatosplenomegaly. (**B**) Coronal T1 WI image following CT examination demonstrated low SI in the diaphyses of the femora extending to extremities. (**C**,**D**) There was high SI in these areas on coronal and axial STIR images with confined edges.

**Figure 2:**
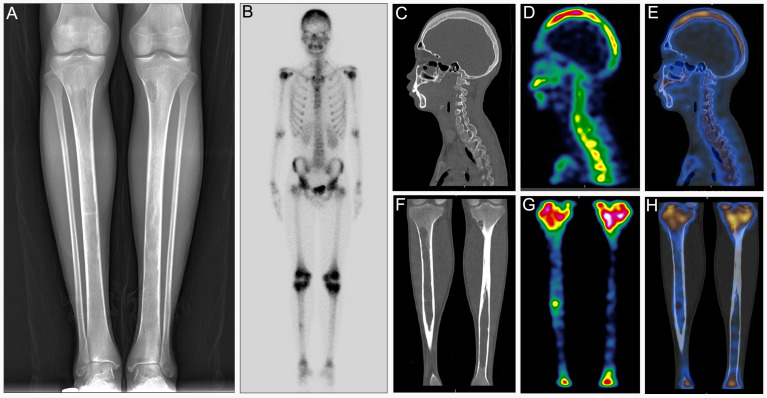
Patient 1: (**A**) Frontal radiograph of left tibiae demonstrated a well-demarcated lytic lesion that did not show sclerotic borders, endosteal erosion or associated expansion of the tibia shaft. (**B**) ${}^{99m}$Tc-MDP bone scan showed abnormal concentration of tracer around the knee joints, in the kull and bilateral humeral heads. (**C**–**E**) CT, SPECT and fusion images of the head. (**F**–**H**) CT, SPECT and fusion images of the both tibiae.

Patient 1 was a 17-year-old man who complained of fatigue, back and lower limbs’ pain for three years. Spleen volume and liver volume of the patient were found to be significantly enlarged upon abdominal computed tomography (CT) (Figure 1A). To explore potential factors causing pain of the lower extremities, the patient was recommended to receive magnetic resonance imaging (MRI) and X-ray examinations. MRI showed bone marrow involvement of the bilateral femora with slightly high signal intensity (SI) on T1-weighted imaging (WI) (Figure 1B) and significantly high SI on T2 fat saturation WI (Figure 1C,D). A frontal radiograph of tibiae demonstrated a well-demarcated lytic lesion in the proximal end of the left tibia without a sclerotic border, endosteal erosion or associated expansion of the tibial shaft. Radiologists supposed that it was a typical nonossifying fibroma. Decreased intensity in the medullary cavities of bilateral tibiae was observed at the same time (Figure 2A). After collecting medical history carefully, we came to know that the patient was from a malaria-endemic area and had acute hemolysis and hematuria after eating fresh fava beans 15 years ago. The patient was further recommended to take a bone scan to exclude other metabolic bone diseases. As a result, ${}^{99m}$Tc-MDP bone scintigraphy showed increased uptake of ${}^{99m}$Tc-MDP in the skull, bilateral humeral heads, bilateral iliac bones, and metaphysis around bilateral knee joints (Figure 2B). Furthermore, ${}^{99m}$Tc-MDP SPECT/CT fusion images demonstrated thickening of cranium (especially in the vault of the skull) and concomitant increased uptake of radiotracer (Figure 2C–E). Notably, medullary intensity of both tibial shafts decreased significantly, and metaphysis and normal remaining bone tissue in the middle of the right shinbone showed increased concentration of ${}^{99m}$Tc-MDP (Figure 2F–H). Considering the patient once had an acute hemolytic reaction triggered by ingestion of fava beans, splenohepatomegalia, imaging features of ${}^{99m}$Tc-MDP SPECT/CT and MRI, a final diagnosis of G-6PD deficiency rather than fibrous dysplasia was made. A subsequent fluorescent spot test confirmed the diagnosis [3].

Hematologic abnormalities of G-6PD deficiency are exceedingly common. Besides ingestion of fava beans, drugs and infection affecting oxidative stress of red blood cells can also trigger manifestations of G-6PD deficiency [1]. This patient subsequently presented with anemia, thrombocytopenia and fatigue. The etiology can be explained by depressed hematopoiesis resulting from substitution of the bone marrow. However, hypersplenism or sequestration within the spleen can be a cause as well. Lieberman et al. have demonstrated that patients with G-6PD deficiency may exhibit splenic accumulation of ${}^{99m}$Tc-MDP without evidence of splenic calcification [2], but there was not any augmented uptake of radiopharmaceutical within the spleen in our case, although physical examination demonstrated a palpable and enlarged spleen further supported by abdominal CT scanning. Despite the delusive image features on MRI, fibrous dysplasia tends to demonstrate marked enhancement throughout the mass on contrast-enhanced MRI [4]. The most effective management strategy for G-6PD deficiency is to prevent hemolysis, by avoiding oxidative stressors (such as drugs and fava beans). Although acute hemolysis in G-6PD deficient individuals is usually short-lived, this patient had severe anemia caused by chronic hemolysis on follow up and then received transfusions of red blood cells after the diagnosis was established.

### 3.2. Gaucher’s Disease (GD)

G-6PD deficiency needs to be differentiated from Gaucher’s disease (GD), where the latter is the most common lysosomal storage disease resulting from accumulation of undegraded glucosylceramide in the reticuloendothelial system of the bone marrow, spleen and liver [5]. Although the clinical manifestations of GD depend on the severity of symptoms and the course of the disease, the most dramatic and debilitating symptoms arise from infiltration of the bone marrow and bone changes, which can lead to pathologic fracture in the late stages.

Patient 2 was a 22-year-old female admitted to our hospital due to suffering from left thigh pain and swelling after an accidental crash. CT scanning revealed altered bone formation, resorption as well as pathologic fracture of the left femur (Figure S1A,B). MRI showed low SI of involved bone marrow of the bilateral femora on T1 WI and heterogenous SI on T2 fat saturation WI (Figure S1C,D). Initial radiological diagnosis of the patient was polyostotic fibrous dysplasia and concomitant pathological fracture of the left femur. The patient then received bone grafting and internal fixation surgery, and postoperative pathology revealed Gaucher’s disease (Figure S1E). Immunohistochemical tests of the tumor tissue disclosed positive PGM-1, Kp-1 (Figure S1F), CD163, periodic acid–Schiff (Figure S1G), iron staining (Figure S1H) and negative S-100, CD5, CD4, SOX10, Langerin, CD1a, CK, EMA and ${\mathrm{BRAF}}^{V600E}$ staining.

One week (Figure S2A,B) and one month (Figure S2C,D) after the surgery, X-ray films were taken to assess the healing process of the fractured bone. Considering the pathology, the patient further received ${}^{18}$F-FDG PET/CT examination to systematically assess the disease burden. Maximum intensity projection (MIP) image with anterior view showed diffuse abnormal FDG uptakes, especially in the bilateral upper and lower extremities. MIP also demonstrated marked hepatosplenomegaly (Figure S3A). The infiltration of the marrow by Gaucher’s cells and fracture of the left femur were confirmed by the fusion images (Figure S3B–D). Notably, CT features in both tibiae of this patient were quite similar to those of patient 1 (Figure S3E and Figure 2F). Replacement of yellow marrow by Gaucher cells in the humerus also led to destruction of normal bone formation and increased glucose metabolism (Figure S3H–J).

Giuliano et al. reported that ${}^{99m}$Tc-Sestamibi uptake reliably identified bone marrow infiltration by Gaucher’s cells, and that scintigraphic score was highly correlated with the severity of bone marrow involvement. The series work by the authors together with others’ indicated the feasibility of directly imaging bone marrow infiltration using ${}^{99m}$Tc-Sestamibi in the early phases of Gaucher’s disease [6,7,8]. However, biodistribution of ${}^{99m}$Tc-Sestamibi did not seem to coincide with that of ${}^{99m}$Tc-MDP [9]. Although both G-6PD deficiency and GD belong to diseases of hematological system and they may have similar patterns on ${}^{99m}$Tc-MDP bone scan and ${}^{18}$F-FDG PET/CT scan, they have totally different features on MRI images, which can help make differential diagnoses. While G-6PD tends to have high SI on both T1- and T2-weighted images, the most frequent MRI abnormality of GD is reduced SI of the bone marrow spaces on the T1- and T2-weighted images (Figure S1C,D) [10]. The decrease in SI may be caused by displacement of the hematopoietic, fat-rich marrow and proinflammatory cytokines in response to pathologic macrophages [11].

### 3.3. Steroid-Induced Osteonecrosis

Osteonecrosis has a large number of etiologies and multifocal osteonecrosis is an uncommon entity usually seen in the clinical setting of corticosteroid administration, connective tissue disorders, dysbarism, hemoglobin patties, arteritis/vasculitis, pancreatitis, Gaucher’s disease, pregnancy and alcohol abuse [12]. We report the development of multiple-site osteonecrosis in a patient with Sjogren syndrome who had received long-term administration of corticosteroid. We know that osteonecrosis is a common finding in a long list of skeletal dysplasia, and the most common in mild and moderate types of lysosomal storage disorders especially in adult patients. Osteoporosis is one of the manifestations of Sjogren syndrome and corticosteroid is an additional factor.

**Figure 3:**
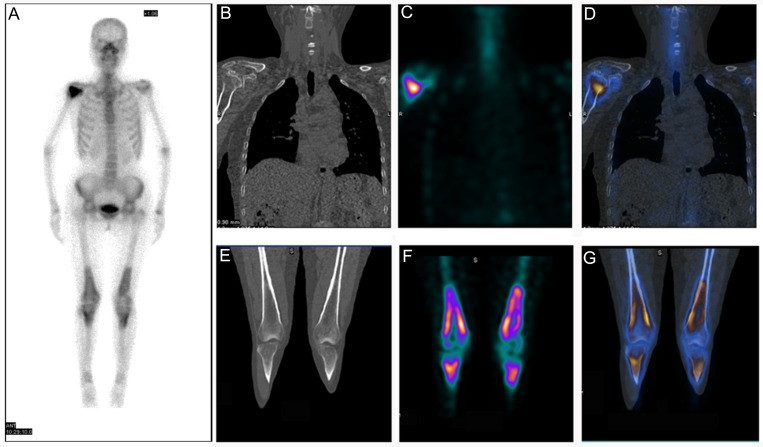
Patient 3: (**A**) ${}^{99m}$Tc-MDP bone scan demonstrated symmetric uptake of radiotracer around both knee joints as well as abnormal tracer uptake in the right humeral head. (**B**–**D**) Coronal CT image revealed “Double line sign” accompanied by increased tracer uptake under articular surface of the corresponding humeral head. (**E**–**G**) CT, SPECT and fusion image of the patient’s bilateral tibiae showed abnormal tracer uptake with seemingly normal CT appearance.

**Figure 4:**
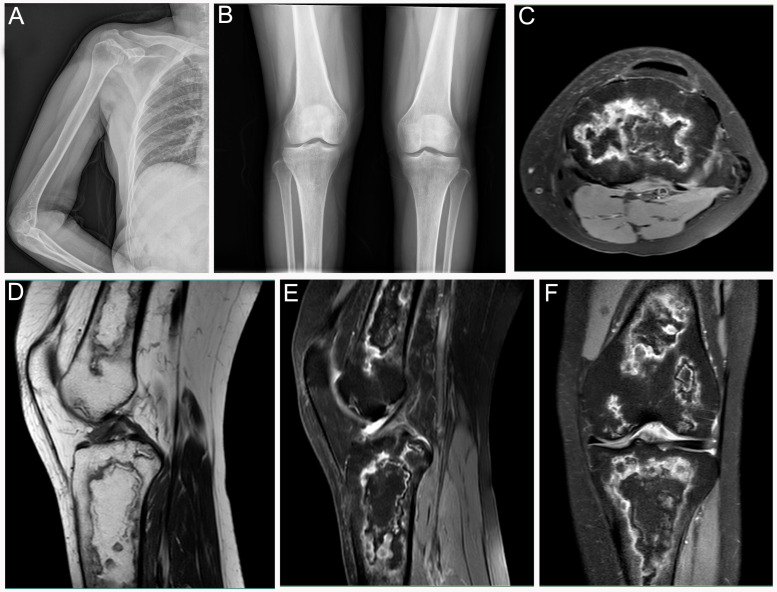
Patient 3: (**A**,**B**) X-ray films of the right upper extremity and knee joints. (**C**–**F**) Sagittal T1-weighted image of left knee showed irregular low-SI areas with low-SI margins. Edema zone surrounding infarcted bone of knee joint presented high SI on transverse, sagittal, and coronal T2-weighted fat-suppressed images.

A 54-year-old female (Patient 3) was admitted to our hospital for evaluation of arthralgia. After initial negative laboratory tests, the patient was recommended to have a ${}^{99m}$Tc-MDP bone scan. The patient had a three-month corticosteroid administration course due to having Sjogren syndrome. Anterior imaging demonstrated increased periarticular tracer uptake around both knee joints and increased uptake in the right humeral head (Figure 3A). ${}^{99m}$Tc-MDP SPECT/CT fusion images revealed abnormal uptake of ${}^{99m}$Tc-MDP in the right humeral head, accompanied by bone destruction (Figure 3B–D). Although SPECT/CT fusion images of the extremities and X-ray film did not find any bone destruction or bone marrow involvement (Figure 3E–G and Figure 4A,B), subsequent MRI of the lower extremities revealed characteristic findings of extensive osteonecrosis. Axial T2-weighted (Figure 4C), sagittal T1-weighted (Figure 4D), sagittal (Figure 4E) and coronal (Figure 4F) T2-weighted images of the right knee demonstrated typical appearances of medullary and corticomedullary infarcts extending to the articular surface.

Early diagnosis of steroid-induced osteonecrosis is of great importance to prevent irreversible bone and joint destruction. It is difficult to judge whether or not increased activity on a plain ${}^{99m}$Tc-MDP bone scan is correlated with revascularization and healing of osteonecrosis [13]. Although it lacks specificity, SPECT/CT may be a useful adjunct in this particular situation by providing anatomic and functional information. MRI has a high sensitivity and specificity in the diagnosis of osteonecrosis, and should be used when steroid-induced osteonecrosis is suspected [13]. If patients have history of steroid intake or chemotherapy containing steroids, patients suffer from bone or joint pains following steroid regimen, or symmetric tracer uptake on the bone scintigraphy, steroid-induced osteonecrosis should be considered and further MRI imaging is definitely needed to consolidate the diagnosis [13,14,15].

### 3.4. Progressive Diaphyseal Dysplasia (PDD)

Progressive diaphyseal dysplasia, or Camurati–Engelmann disease (CED), is a rare autosomal dominant skeletal dysplasia caused by mutations in the transforming growth factor-b1 (TGF β 1) gene [16]. TGF β 1 interferes with the bone remodeling process by stimulating bone formation and suppressing bone resorption under physiologic conditions, therefore, mutations in the TGF β 1 gene often lead to increased intramembranous bone formation. PDD is a rare genetic disease with variable clinical manifestations, and needs to be considered in the differential diagnosis of nonspecific limb pain and waddling gait in all young individuals.

**Figure 5:**
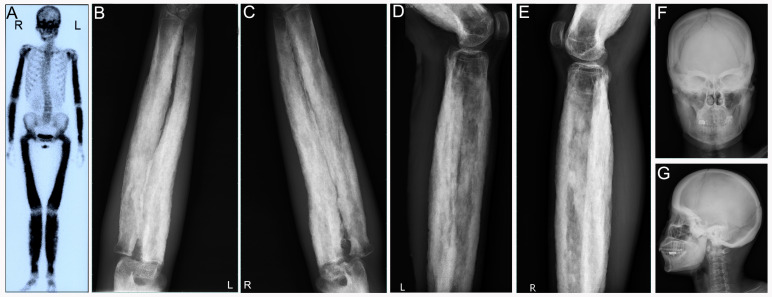
Patient 4: (**A**) ${}^{99m}$Tc-MDP bone scan demonstrated symmetric uptake of radiotracer mainly in the skull, upper and lower extremities. Radiographs of both upper limbs (**B**,**C**) and lower limbs (**D**,**E**) revealed thickened cortices and narrowed medullary space. (**F**,**G**) X-ray films of anteroposterior position and lateral position showed the skull of the patient was thickened as well.

**Figure 6:**
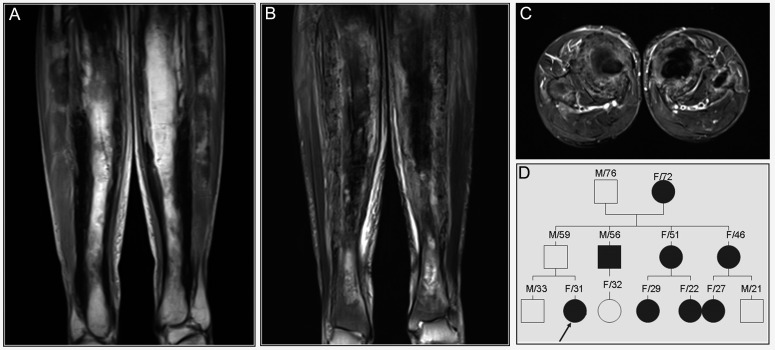
Patient 4: (**A**) MRI images of the both femora showed thickened cortices with low SI on T1 WI (**A**) and heterogenous SI on T2 fat suppression WI (**B**,**C**). (**D**) Family pedigree of the patient’s family, indicating an autosomal dominant mode of inheritance. Arrow indicates the index case, dark circle is affected individual, and gray square is asymptomatic carrier.

A reported case (Patient 4) presented at 30 years of age with complaints of pain over both lower limbs and increasing difficulty in walking. Laboratory studies showed that serum calcium, phosphorus and PTH were within the normal range. Alkaline phosphatase was elevated (217 U/L, normal range: 15–112 U/L). Her CTX-I was elevated as well (715 ng/L, normal range: 513 ng/L), with decreased hemoglobin (87 g/L, normal range: 113–142 g/L) and increased blood sedimentation rate. A ${}^{99m}$Tc-MDP bone scan revealed increased tracer uptake in the extremities and skull. The rest of the skeleton was relatively spared. Radiographs of bilateral lower limbs and upper limbs revealed thickened cortices with irregular endosteal, diaphyseal sclerosis and narrowing marrow cavity (Figure 5B–E). The skull of the patient was thickened, and sclerosis of the tympanic portion of the skull base was observed at the same time (Figure 5F,G). MRI images of the both femora showed thickened cortices with low SI on T1 WI (Figure 6A) and heterogenous SI on T2 fat suppression WI (Figure 6B,C). On detailed evaluation, there was a strong family history of bone disorders, indicating an autosomal dominant mode of inheritance (Figure 6D). The onset of symptoms was noted after puberty in affected family members. The clinical findings, characteristic radiological appearances and family history led to a diagnosis of PDD.

Camurati first suggested the familial nature of PDD in 1922, and subsequently, Engelmann reported a single case with muscular wasting and marked bone involvement in 1929 [16]. The typical clinical features include leg pain, muscle weakness, waddling gait, hearing loss, and easy fatigability after the onset of puberty. Although quite rare, systemic manifestations of anemia, leucopenia, and hepatosplenomegaly can appear [17]. The patient reported here suffered from anemia due to the severe involvement of the marrow cavity. Generally, uptake of ${}^{99m}$Tc-MDP in the longitudinal bone cortices was consistent with sclerosing dysplasia on bone X-ray films [18,19,20,21].

### 3.5. Erdheim–Chester Disease (ECD)

Erdheim–Chester disease (ECD) is a rare non–Langerhans cell histiocytosis characterized by infiltration of tissues by foamy histiocytes [22]. By June 2014, a total of 448 ECD cases had been reported [23]. This disease has a broad spectrum of clinical manifestations, and generally presents with bone pain and atypically with neurological system involvement or renal failure.

The first case reported (Patient 5) was a 51-year-old male patient who suffered from renal failure in 2011 and skin pigmentation of the anterior chest wall in 2012. Since 2013, the patient has suffered from increasing bone pain of the lower extremities. A timely ${}^{99m}$Tc-MDP bone scan showed abnormal symmetrical concentration of radiotracer in the distal femora and proximal tibiae (Figure S4A). The patient further underwent a CT scan followed by CT-guided tissue biopsy of the distal left femur. CT showed diffuse osteosclerosis in the distal femur and the corresponding marrow cavity of the femur was narrowed (Figure S4B,C). Final pathology of the patient was Erdheim–Chester disease characterized by positive CD68, CD138, Lys, Ki67 (10%) staining and negative S-100, and CD1a staining. The final diagnosis of the patient was based on radiographic findings of medullary sclerosis confined to the appendicular skeleton, ${}^{99m}$Tc-MDP bone scan and pathologic features.

**Figure 7:**
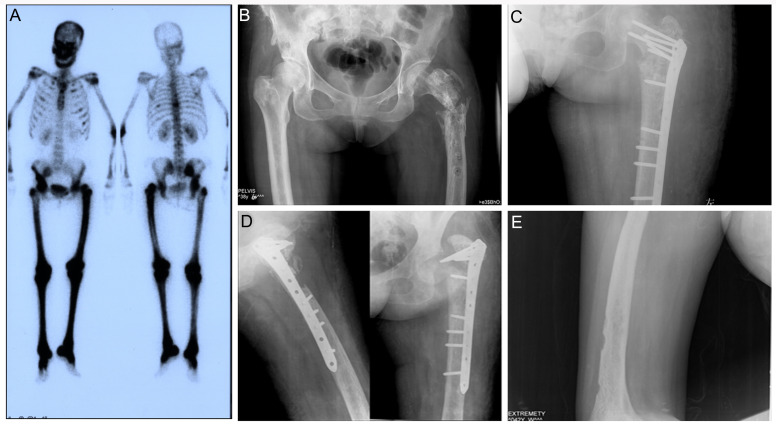
Patient 6: (**A**) ${}^{99m}$Tc-MDP bone scan showed increased uptake in the metaphyses and diaphyses of long bones, iliac bones and ribs. (**B**) Subsequent X-ray examination of pelvis revealed fracture near greater trochanter of the left femur. Postoperative X-ray examination (**C**) and X-ray examination performed 3 years after the internal fixation surgery (**D**) demonstrated disunion of the fractured femur. (**E**) X-ray of the left femur revealed diffuse osteosclerosis with focal osteolysis in the diaphysis and metaphysis of the distal right femur.

**Figure 8:**
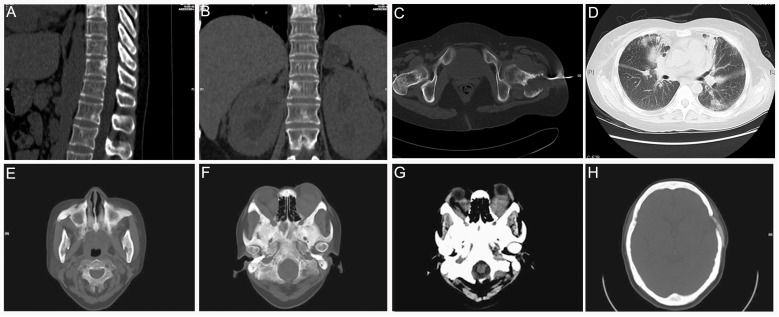
Patient 6: (**A**,**B**) Vertebrae involvement was detected on sagittal and coronal multiplanar reconstruction CT. (**B**) Enlargement and nephrydrosis of bilateral kidneys were also observed. (**C**) Axial CT shows biopsy of the left proximal femur. (**D**) Axial CT shows progressive pulmonary interstitial fibrosis. (**E**,**F**) Axial CT scans showed bilateral osteosclerosis encompassing the sphenoid sinuses. (**G**) Exophthalmos caused by bilateral retro-ocular intraconal infiltration. (**H**) Corresponding axial CT shows biopsy of the left temporal bone.

**Figure 9:**
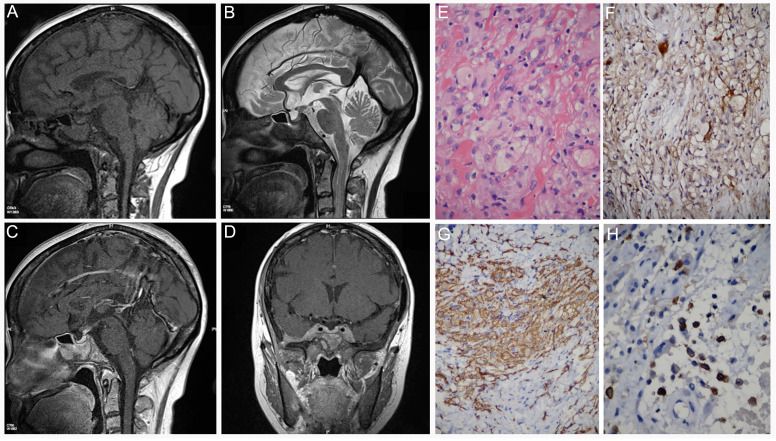
Patient 6: (**A**) Sagittal T1-weighted image showed marked thickening of the mucoperiosteal lining in the sphenoid sinus and absence of normal bright spot signal in the posterior pituitary. (**B**) Sagittal T2-weighted image showed a sellar arachnoid cyst and pituitary gland was compressed to the bottom of the sellar. (**C**) Gadolinium-enhanced sagittal T1-weighted image showed no enhancement of the sellar arachnoid cyst. (**D**) Coronal T1-weighted gadolinium-enhanced MRI showed diffuse enhancement and thickening of the skull. Biopsy samples obtained from left femur and left temporal bone showed similar infiltration of numerous histiocytes. Light microscopy showed foamy histiocytes, eosinophils, and lymphocytes ((**E**), ×100). The histiocytes were S-100-positive ((**F**), ×100), CD163-positive ((**G**), ×100) and CD1a-negative ((**H**), ×100).

We have encountered another two ECD cases and both patients suffered from orbital and sphenoid bone involvement and subsequent visual extinction. A ${}^{99m}$Tc-MDP bone scan of one of the two patients is shown in Figure 7A. The patient (Patient 6) was a 38-year-old woman presenting with a fracture of the left proximal femur after a minor injury. She had been hospitalized in the endocrinology department for central diabetes insipidus and was being treated with desmopressin. She recently complained of coughing and dyspnea. In addition, her vision deteriorated and her eyes protruded to the point that she could not close her eyelids. A ${}^{99m}$Tc-MDP bone scan revealed diffuse uptake of radiotracer in the skull and lower extremities (Figure 7A). Abnormal concentration of radiotracer was also observed in bilateral humeral heads and iliac bones. A radiograph of her pelvis showed pathologic fracture of the left proximal femur, and both lytic and sclerotic lesions could be found at the right hip bone (Figure 7B). The patient then received internal fixation of the fractured femur (Figure 7C) and X-ray performed 3 years after surgery indicated that the fracture did heal (Figure 7D). X-ray also revealed diffuse osteosclerosis with focal osteolysis in the diaphysis and metaphysis of the distal right femur (Figure 7E). The multifocal nature of the patient’s osseous lesions prompted a complete diagnostic workup with CT and MRI followed by tissue biopsy from two sites: the left femur and the left temporal bone. Sagittal and coronal multiplanar reconstruction CT showed lytic and sclerotic lesions in multiple vertebral bodies and enlargement of bilateral kidneys (Figure 8A–C). In addition, axial CT showed progressive pulmonary interstitial fibrosis (Figure 8D). Cranial CT scans showed bilateral osteosclerosis encompassing the sphenoid sinuses (Figure 8E,F) and exophthalmos caused by bilateral retroocular infiltration (Figure 8G). The sagittal T1 WI showed marked thickening of the mucoperiosteal lining in the sphenoid sinus and absence of a normal bright spot signal in the posterior pituitary gland (Figure 9A). The sagittal T2 WI showed a sellar arachnoid cyst, and the pituitary gland was compressed to the bottom of the sellar (Figure 9B). The gadolinium-enhanced sagittal T1 WI showed no enhancement of the sellar arachnoid cyst (Figure 9C). Coronal T1-weighted gadolinium-enhanced MRI showed diffuse enhancement and thickening of skull (Figure 9D). Microscopic examination of the biopsy specimens showed an infiltrate of foamy histiocytes with bland nuclei admixed with Touton giant cells, lymphocytes, and eosinophils (Figure 9E). Immunocytochemistry stained positive for S-100 protein (Figure 9F) and CD163 (Figure 9G) and negative for CD1a (Figure 9H). Positive staining of CD163 excluded the diagnosis of Langerhans cell histiocytosis.

The third patient (Patient 7) was a 56-year-old female who complained of interrupted fevering and paining of bilateral knee joints since 2014. The patient had Sjogren’s syndrome and usually took hydroxychloroquine and prednisone when the disease was active. The patient also suffered from diabetes and anxious depression for 3 years. Because of increasing pain and stiffness of the knee joints and right finger joints, she was admitted to department of osteoporosis in our hospital for further evaluation. The results of extensive serum laboratory analyses were otherwise unremarkable. Plain film showed abnormal density of sella turcica and basalis (Figure S5A,B), sclerosis and narrowing of marrow cavity of distal femora and proximal tibiae (Figure S5C,D) as well as bilateral ulnar bones and radial bones (Figure S5E). MRI imaging of lower extremities showed patchy diseased areas in the distal femora and proximal tibiae, with low SI on T1 WI (Figure S6A) and high SI on T2 fat suppression WI (Figure S6B). Notably the diseased areas were enhanced significantly (Figure S6C). The pituitary was normal on brain MRI imaging. The patient further underwent ${}^{18}$F-FDG PET/CT scanning to exclude tumorous diseases. MIP (Figure S7A) and fusion images disclosed increased uptake of ${}^{18}$F-FDG, mainly in the bilateral distal femora and proximal tibiae. Increased concentration of radiotracer around the knee joints coincided with thickened cortices and narrowed marrow cavity (Figure S7B–G). From a radiological aspect we gave a possible diagnosis of ECD considering its radiological features and multiple-organ involvement. Subsequent histopathological diagnosis of ECD by bone and bone marrow biopsy was established. Clinical physicians provided four treatment options: steroid, interferon, chemotherapy and cladribine [24]. The patient and family chose interferon treatment considering the adverse effects of other options.

ECD is a rare disease first described by Jacob Erdheim and William Chester in 1930. The disease is characterized by tissue infiltration by foamy histiocytes, typically in the long bone marrow, but also in numerous other organs [25]. Common medical complications include progression to respiratory, renal, skin and cardiac failure [26]. Neurological symptoms represent a prominent feature of ECD and occur in approximately 50% of patients during the course of the disease [23]. One of our ECD patients suffered from exophthalmos and diabetes insipidus, which was the most common manifestation of neuro-ECD and was associated with thickening of the pituitary stalk, alteration of the brightness of the hypophysis or pituitary infiltration. Another patient suffered from neuropsychiatric symptoms (depression), which was observed in less than 10% of patients [23]. The diagnosis relies exclusively upon correlation of the radiographic and pathologic findings because of its nonspecific clinical presentation. Conventional radiography of ECD typically shows a bilateral symmetric pattern of medullary sclerosis involving the diametaphyses of the lower extremities with sparing of the epiphysis [27]. Radiological features of ECD are believed to be consistent with that of ${}^{99m}$Tc-MDP bone scans [26,28,29,30,31]. Despite similar symmetrical patterns on the ${}^{99m}$Tc-MDP plain film, ECD tends to involve extremities or metaphyses, while PDD impinge diaphyses more often. However, the rarity of the disease hinders the diagnosis and necessitates extensive imaging examinations, in which bone scans, CT and MRI of the central nervous system (CNS) are essential components. Beylergil et al. reported that ${}^{18}$F-FDG PET/CT may provide additional value in the assessment of orbital involvement in ECD [32]. Previous studies have reported that PET scanning had high specificity for brain ECD lesions and should be considered for the initial assessment and follow-up of ECD patients displaying pituitary involvement [23,33]. Here, we further demonstrated that ${}^{18}$F-FDG PET/CT imaging can provide similar diagnostic value when assessing ECD burden without pituitary involvement.

### 3.6. Langerhans Cell Sarcoma

Langerhans cell tumors are generally classified into Langerhans cell histiocytosis (LCH) and Langerhans cell sarcoma (LCS), and while the former is a benign entity the latter is a rare and malignant tumor of Langerhans cells. Up to now, there are only 66 LCS cases reported in the literature [34]. Given its rarity, there is a lack of evidence regarding the medical imaging patterns for this condition.

**Figure 10:**
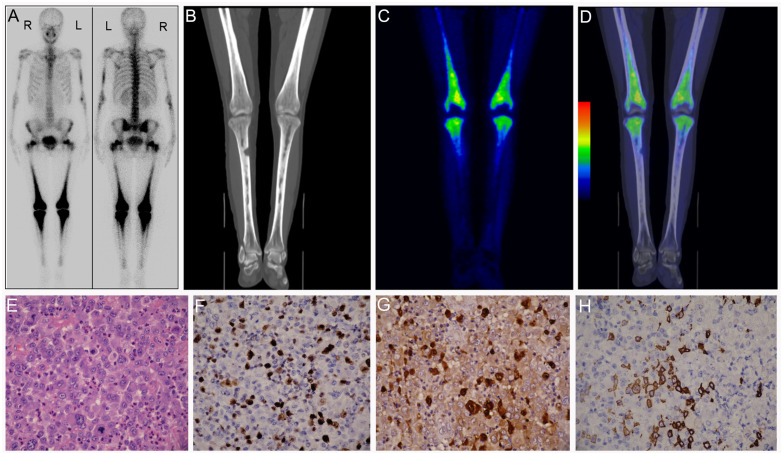
Patient 8: ${}^{99m}$Tc-MDP bone scan (**A**) and ${}^{18}$F-FDG-PET/CT scan (**B**–**D**) of the patient. HE staining of the tumor tissue showed that tumor cells have malignant cytologic features and a high mitotic rate (**E**). Immunohistochemistry showed that tumor cells expressed Ki-67 (**F**), S-100 (**G**) and CD1a (**H**).

The last patient (Patient 8) was a 47-year-old female patient initially presenting with lower leg pain. Previously, she had received surgery on the right tibia because of suspicious chronic osteomyelitis. A ${}^{99m}$Tc-MDP bone scan of the patient revealed diffused and symmetric uptake of radiotracer in the distal femoral bones and proximal shin bones, and increased concentration of radiotracer was also found in her hip joints, sacroiliac joints, elbow joints as well as in the middle part of the left humerus (Figure 10A). Subsequent ${}^{18}$F-FDG PET/CT further reported abnormal glucose metabolism in bone marrow of the corresponding diseased areas with a SUVmax of 4.5 (Figure 10B–D). Our nuclear medicine physicians consistently diagnosed the case as a typical ECD. Based on her medical history and these imaging findings, pathologists suggested a consultation of her previous surgical specimens. Beyond our imagination, final pathological results showed malignant cytological features, atypia and mitoses (Figure 10E), and positive immunohistochemical staining for Ki-67 (40%, Figure 10F), S-100 (Figure 10G), Langerin and CD1a (Figure 10H) protein, which were confirmatory of LCS. The expression of histiocytic marker lysozyme, Kp1, CD163, PGM1 were also positive for this patient. Histopathology also found that the tumor infiltrated cortical plate and adjacent soft tissue. Currently the patient is receiving chemotherapy, but the optimal treatment strategy and efficacy is still in debate [35,36].

LCS can present de novo or progress from antecedent LCH. Skin, lymph nodes, spleen, liver, bone marrow, thymus, lung, and kidney have been frequently involved [37]. It is notable that LCS can undergo leukemic transformation [35,38,39,40]. Although our patient did not have bone marrow aspiration, abnormal laboratory results or splenohepatomegalia, her imaging features upon ${}^{99m}$Tc-MDP bone scan and ${}^{18}$F-FDG PET/CT resembled that of the above-mentioned hematological diseases. LCS is unfamiliar to nuclear medicine physicians, and to our best knowledge, this is the first time that the imaging features of LCS on both ${}^{99m}$Tc-MDP bone scan and ${}^{18}$F-FDG PET/CT are reported. However, from this specific case we come to know that differential diagnosis between LCS and ECD is quite difficult from a medical imaging perspective.

### 3.7. Others

Besides those rare diseases involving metaphyses/extremities of lower extremities, fibrous dysplasia, hyperparathyroidism and other metabolic bone diseases are of relatively higher incidence and may present similar bone scan patterns. Fibrous dysplasia (FD), which accounts for about 2–3% of bone-derived tumors, is a benign bone lesion characterized by the replacement of normal bone structure with abnormal fibro-osseous connective tissue [41]. It has been reported that monostotic FD accounted for 70% and polyostotic FD accounted for 30% in a Chinese population [42]. Diagnosis of fibrous dysplasia is based on clinical examination, radiographic and histopathological findings. We previously studied the bone scan findings of 42 cases of FD and found that the extremities and ribs are the most common sites of involvement in FD, and further illustrated that Paget’s disease resembles FD on bone scans but the former generally contains osteolytic, osteogenic, sclerotic features on X-ray/CT [43].

Hyperparathyroidism, especially primary hyperparathyroidism (PHPT), is a frequent and potentially debilitating endocrine disorder. In up to 85% of clinical cases, PHPT is characterized by the autonomous overproduction of parathyroid hormone (PTH) by a solitary parathyroid adenoma [44]. We previously reported that ${}^{99m}$Tc-MIBI scintigraphy and ${}^{99m}$Tc-MDP bone scintigraphy are closely correlated with tumor diameter and PTH level [45]. Therefore, combination of laboratory tests, ${}^{99m}$Tc-MIBI planar or SPECT/CT fusion images and ${}^{99m}$Tc-MDP bone scintigraphy is of great value to make a differential diagnosis. In addition, super-bone-scans are observed in tumorous diseases such as agnogenic myeloid metaplasia [46], gastric cancer [47,48,49], intracranial glioma [50], prostate cancer [51,52], breast cancer [49] and even recurrent syncope [53]. In some of these cases, superscans on bone scintigraphy were caused by concomitant metastatic and metabolic bone diseases. Therefore, caution should be given when interpreting a bone scintigraphy in patients with known malignancy because the appearance of metabolic disease may obscure recognition of osseous metastases.

## 4. Conclusions

Here, we show that when symmetrical uptake of radiotracer appears on ${}^{99m}$Tc-MDP bone scintigraphy or ${}^{18}$F-FDG PET/CT scans, other potential causes except for FD, hyperparathyroidism and metabolic bone diseases should be considered. Together with examinations including laboratory tests, X-ray, CT, MRI and medical history, it is possible to draw a correct diagnosis, despite the rare properties of these diseases. SPECT/CT is a complimentary procedure and not mandatory to every case. It can be suggested after thorough clinical and conventional phenotypic characterizations. However, whole-body bone scanning with or without SPECT/CT might show us unusual findings and help us to understand the extent of the disease.

## Data Availability

Not applicable.

## Supplementary Materials

The following supporting information can be downloaded at: https://www.mdpi.com/article/10.3390/diagnostics12040910/s1, Figure S1: Patient 2: (A,B) Initial CT examination revealed dereased intensity of the involved medullary canal and pathological fracture of left femur. (C,D) Subsequent coronal T1 WI showed areas of low SI within the medullary cavity of both femora, the corresponding areas show heterogeneous low SI on the coronal short tau inversion recovery (STIR). Postoperative hematoxylin and eosin staining of the patient revealed Gaucher’s disease (E). Immunohistochemical test of the tumor tissue disclosed positive Kp-1 (F), periodic acid–Schiff (G) and iron staining (H), Figure S2: (A,B) Postoperative radiograph of the pelvis and femora, demonstrating decreased intensity and disformation of both femora. (C,D) plane radiograph taken on follow-up (one month after the surgery) indicated the disunion of the fractured femur, Figure S3: Patient 2: (A) Postoperative systematic assessment of disease burden using ${}^{18}$F-FDG PET/CT. Maximum intensity projection demonstrated diffuse uptake of tracer in the extremities, axial skeletons, iliac bones, liver and spleen. Hepatosplenomegaly was also observed. (B–G) CT, PET and fusion images of both femora and tibiae. (H–J) CT, PET and fusion images of bilateral humeral bones, Figure S4: Patient 5: (A) ${}^{99m}$Tc-MDP bone scan demonstrated symmetric uptake of radiotracer in the distal femora, tibial metaphyses. (B,C) Coronal and sagittal CT showed osteosclerosis and narrowed marrow cavity in the distal femur, Figure S5: Patient 7: Anteroposterior position (A) and lateral position (B) plain film of the skull. Femora and tibiae of the patient were affected. Diaphyseal osteosclerosis of the distal femur (C), proximal tibia (D), ulna and radius (E) as well as narrowing of the correponding marrow cavities were observed on X-ray plain film, Figure S6: Patient 7: MRI imaging of the lower extremities indicated that bilateral patchy diseases appeared as low SI on T1 WI (A) and heterogeneous high SI on T2 WI (B). The MRI documented a homogeneous intense enhancement of the patchy diseases after gadolinium administration (C), Figure S7: Patient 7: (A) ${}^{18}$F-FDG-PET/CT demonstrated high glucose avidity of the metaphyses of lower extremities with a SUVmax of 2.4. (B–D) Cortical substance of bilateral distal femora thickened and corresponding marrow cavities narrowed accordingly, accompanied by increased uptake of radiotracer. (E–G) similar changes regarding bone formation and glocuse metabolism were observed in both tibiae.

## References

[1] Cappellini M.D., Fiorelli G. (2008). Glucose-6-phosphate dehydrogenase deficiency. Lancet.

[2] Lieberman C.M., Hemingway D.L. (1979). Splenic visualization in a patient with glucose-6-phosphate dehydrogenase deficiency. Clin. Nucl. Med..

[3] Nadarajan V., Shanmugam H., Sthaneshwar P., Jayaranee S., Sultan K.S., Ang C., Arumugam S. (2011). Modification to reporting of qualitative fluorescent spot test results improves detection of glucose-6-phosphate dehydrogenase (G6PD)-deficient heterozygote female newborns. Int. J. Lab. Hematol..

[4] Qu N., Yao W., Cui X., Zhang H. (2015). Malignant transformation in monostotic fibrous dysplasia: Clinical features, imaging features, outcomes in 10 patients, and review. Medicine.

[5] Mariani G., Perri M., Minichilli F., Ortori S., Linari S., Giona F., Di Rocco M., Cappellini M.D., Guidoccio F., Erba P.A. (2016). Standardization of MRI and Scintigraphic Scores for Assessing the Severity of Bone Marrow Involvement in Adult Patients With Type 1 Gaucher Disease. AJR Am. J. Roentgenol..

[6] Mariani G., Filocamo M., Giona F., Villa G., Amendola A., Erba P., Buffoni F., Copello F., Pierini A., Minichilli F. (2003). Severity of bone marrow involvement in patients with Gaucher’s disease evaluated by scintigraphy with ^99m^Tc-sestamibi. J. Nucl. Med..

[7] Erba P.A., Minichilli F., Giona F., Linari S., Dambrosia J., Pierini A., Filocamo M., Di Rocco M., Buffoni F., Brady R.O. (2013). ^99m^Tc-sestamibi scintigraphy to monitor the long-term efficacy of enzyme replacement therapy on bone marrow infiltration in patients with Gaucher disease. J. Nucl. Med..

[8] Aharoni D., Krausz Y., Elstein D., Hadas-Halpern I., Zimran A. (2002). Tc-99m sestamibi bone marrow scintigraphy in Gaucher disease. Clin. Nucl. Med..

[9] Mariani G., Molea N., La Civita L., Porciello G., Lazzeri E., Ferri C. (1996). Scintigraphic findings on ^99m^Tc-MDP, ^99m^Tc-sestamibi and ^99m^Tc-HMPAO images in Gaucher’s disease. Eur. J. Nucl. Med..

[10] Katz R., Booth T., Hargunani R., Wylie P., Holloway B. (2011). Radiological aspects of Gaucher disease. Skeletal Radiol..

[11] Allen M.J., Myer B.J., Khokher A.M., Rushton N., Cox T.M. (1997). Pro-inflammatory cytokines and the pathogenesis of Gaucher’s disease: Increased release of interleukin-6 and interleukin-10. QJM Int. J. Med..

[12] Ziraldo L., O’Connor M.B., Blake S.P., Phelan M.J. (2011). Osteonecrosis following alcohol, cocaine, and steroid use. Subst. Abuse.

[13] Jones D.N. (1994). Multifocal osteonecrosis following chemotherapy and short-term corticosteroid therapy in a patient with small-cell bronchogenic carcinoma. J. Nucl. Med..

[14] Thornton M.J., O’Sullivan G., Williams M.P., Hughes P.M. (1997). Avascular necrosis of bone following an intensified chemotherapy regimen including high dose steroids. Clin. Radiol..

[15] Flouzat-Lachaniette C.H., Younes C., Delblond W., Dupuy N., Hernigou P. (2012). The natural progression of adult elbow osteonecrosis related to corticosteroid treatment. Clin. Orthop. Relat. Res..

[16] Janssens K., Vanhoenacker F., Bonduelle M., Verbruggen L., Van Maldergem L., Ralston S., Guanabens N., Migone N., Wientroub S., Divizia M.T. (2006). Camurati-Engelmann disease: Review of the clinical, radiological, and molecular data of 24 families and implications for diagnosis and treatment. J. Med. Genet..

[17] Crisp A.J., Brenton D.P. (1982). Engelmann’s disease of bone—A systemic disorder?. Ann. Rheum. Dis..

[18] Momose M., Yoshida K., Yanagisawa S., Kadoya M. (2008). Camurati-Engelmann disease on a ^99m^Tc-HMDP bone scan. Eur. J. Nucl. Med. Mol. Imaging.

[19] Shier C.K., Krasicky G.A., Ellis B.I., Kottamasu S.R. (1987). Ribbing’s disease: Radiographic-scintigraphic correlation and comparative analysis with Engelmann’s disease. J. Nucl. Med..

[20] Kumar B., Murphy W.A., Whyte M.P. (1981). Progressive diaphyseal dysplasia (Engelmann disease(: Scintigraphic-radiographic-clinical correlations. Radiology.

[21] Damia Ade B., Moron C.C., Perez P.A., Molina T.C., Mora M.M., Fatou A.F., Montano J.C. (2010). Bone scintigraphy in Engelmann-Camurati disease. Clin. Nucl. Med..

[22] Veyssier-Belot C., Cacoub P., Caparros-Lefebvre D., Wechsler J., Brun B., Remy M., Wallaert B., Petit H., Grimaldi A., Wechsler B. (1996). Erdheim-Chester disease. Clinical and radiologic characteristics of 59 cases. Medicine.

[23] Cives M., Simone V., Rizzo F.M., Dicuonzo F., Cristallo Lacalamita M., Ingravallo G., Silvestris F., Dammacco F. (2015). Erdheim-Chester disease: A systematic review. Crit. Rev. Oncol. Hematol..

[24] Arnaud L., Hervier B., Neel A., Hamidou M.A., Kahn J.E., Wechsler B., Perez-Pastor G., Blomberg B., Fuzibet J.G., Dubourguet F. (2011). CNS involvement and treatment with interferon-alpha are independent prognostic factors in Erdheim-Chester disease: A multicenter survival analysis of 53 patients. Blood.

[25] Strouse P.J., Ellis B.I., Shifrin L.Z., Shah A.R. (1992). Case report 710: Symmetrical eosinophilic granuloma of the lower extremities (proven) and Erdheim-Chester disease (probable). Skeletal Radiol..

[26] Shah M.V., Go R.S. (2015). Erdheim-Chester Disease. Mayo Clin. Proc..

[27] Resnick D., Greenway G., Genant H., Brower A., Haghighi P., Emmett M. (1982). Erdheim-Chester disease. Radiology.

[28] Nunez R., Tronco G.G., Rini J.N., Hofman J., Amoashiy M., Bhuiya T., Palestro C.J. (2005). Radionuclide bone imaging in Erdheim-Chester disease. Clin. Nucl. Med..

[29] Canbaz F., Dabak N., Baris S., Selcuk M.B. (2005). Erdheim-Chester disease: ^99m^Tc-MDP bone scan provides the diagnosis. Eur. J. Nucl. Med. Mol. Imaging.

[30] Namwongprom S., Nunez R., Kim E.E., Macapinlac H.A. (2007). Tc-99m MDP bone scintigraphy and positron emission tomography/computed tomography (PET/CT) imaging in Erdheim-Chester disease. Clin. Nucl. Med..

[31] Spyridonidis T.J., Giannakenas C., Barla P., Apostolopoulos D.J. (2008). Erdheim-Chester disease: A rare syndrome with a characteristic bone scintigraphy pattern. Ann. Nucl. Med..

[32] Beylergil V., Carrasquillo J.A., Hyman D.M., Diamond E.L. (2014). Visualization of orbital involvement of Erdheim-Chester disease on PET/CT. Clin. Nucl. Med..

[33] Arnaud L., Malek Z., Archambaud F., Kas A., Toledano D., Drier A., Zeitoun D., Cluzel P., Grenier P.A., Chiras J. (2009). 18F-fluorodeoxyglucose-positron emission tomography scanning is more useful in followup than in the initial assessment of patients with Erdheim-Chester disease. Arthritis Rheum..

[34] Howard J.E., Dwivedi R.C., Masterson L., Jani P. (2015). Langerhans cell sarcoma: A systematic review. Cancer Treat. Rev..

[35] Sumida K., Yoshidomi Y., Koga H., Kuwahara N., Matsuishi E., Karube K., Oshima K., Gondo H. (2008). Leukemic transformation of Langerhans cell sarcoma. Int. J. Hematol..

[36] Howard J.E., Masterson L., Dwivedi R.C., Jani P. (2016). Langerhans cell sarcoma of the head and neck. Crit. Rev. Oncol. Hematol..

[37] Bohn O.L., Ruiz-Arguelles G., Navarro L., Saldivar J., Sanchez-Sosa S. (2007). Cutaneous Langerhans cell sarcoma: A case report and review of the literature. Int. J. Hematol..

[38] Henderson D.W., Sage R.E. (1973). Malignant histiocytosis with eosinophilia. Cancer.

[39] Kawase T., Hamazaki M., Ogura M., Kawase Y., Murayama T., Mori Y., Nagai H., Tateno M., Oyama T., Kamiya Y. (2005). CD56/NCAM-positive Langerhans cell sarcoma: A clinicopathologic study of 4 cases. Int. J. Hematol..

[40] Imamura M., Sakamoto S., Hanazono H. (1971). Malignant histiocytosis: A case of generalized histiocytosis with infiltration of Langerhans’ granule-containing histiocytes. Cancer.

[41] DiCaprio M.R., Enneking W.F. (2005). Fibrous dysplasia. Pathophysiology, evaluation, and treatment. J. Bone Jt. Surg. Am..

[42] Cheng J., Wang Y., Yu H., Wang D., Ye J., Jiang H., Wu Y., Shen G. (2012). An epidemiological and clinical analysis of craniomaxillofacial fibrous dysplasia in a Chinese population. Orphanet J. Rare Dis..

[43] Zhibin Y., Quanyong L., Libo C., Jun Z., Hankui L., Jifang Z., Ruisen Z. (2004). The role of radionuclide bone scintigraphy in fibrous dysplasia of bone. Clin. Nucl. Med..

[44] Wei W.J., Shen C.T., Song H.J., Qiu Z.L., Luo Q.Y. (2015). Comparison of SPET/CT, SPET and planar imaging using ^99m^Tc-MIBI as independent techniques to support minimally invasive parathyroidectomy in primary hyperparathyroidism: A meta-analysis. Hell. J. Nucl. Med..

[45] Qiu Z.L., Wu B., Shen C.T., Zhu R.S., Luo Q.Y. (2014). Dual-phase (99m)Tc-MIBI scintigraphy with delayed neck and thorax SPECT/CT and bone scintigraphy in patients with primary hyperparathyroidism: Correlation with clinical or pathological variables. Ann. Nucl. Med..

[46] Pour M.C., Simon-Corat Y., Horne T. (2004). Diffuse increased uptake on bone scan: Super scan. Semin. Nucl. Med..

[47] Omi R., Hatori M., Sano H., Watanabe K., Watanabe M., Kokubun S. (2004). Super bone scan due to bone marrow metastases appearing 19 years after surgery for early gastric cancer—A case report. Upsala J. Med. Sci..

[48] Kanazawa K., Isozaki E. (2009). “Super bone scan” in a case of diffuse bone marrow metastasis of gastric adenocarcinoma. Intern. Med..

[49] Lin C.Y., Chen Y.W., Chang C.C., Yang W.C., Huang C.J., Hou M.F. (2013). Bone metastasis versus bone marrow metastasis? Integration of diagnosis by (18)F-fluorodeoxyglucose positron emission/computed tomography in advanced malignancy with super bone scan: Two case reports and literature review. Kaohsiung J. Med. Sci..

[50] Shinya T., Akaki S., Ogata T., Sato S., Kanazawa S. (2007). Super scan in a patient with diffuse bone metastases from intracranial glioma. Clin. Nucl. Med..

[51] Liu Y. (2011). Super-superscan on a bone scintigraphy. Clin. Nucl. Med..

[52] Seo Y., Shuke N., Yamamoto W., Usui K., Aburano T. (1999). Sub-super bone scan caused by bone marrow involvement of prostate cancer. Ann. Nucl. Med..

[53] Khoury J., Jerushalmi J., Cohen H.I., Loberant N., Zaina A. (2003). Super scan leading to definitive diagnosis in a patient with recurrent syncope. Clin. Nucl. Med..

