# Supplementary material for: Differential Diagnosis of Rare Diseases Involving Bilateral Lower Extremities with Similar 99mTc-MDP Bone Scan Patterns: Analysis of a Case Series
Source: Diagnostics (Basel). 2022 Apr 6;12(4):910. doi: 10.3390/diagnostics12040910 (PMC9031204; doi:10.3390/diagnostics12040910)
Supplement: Supplementary file 1 [file diagnostics-12-00910-s001.zip › diagnostics-1630214-supplementary.pdf]

Supplemental Figures and Figure Legends:

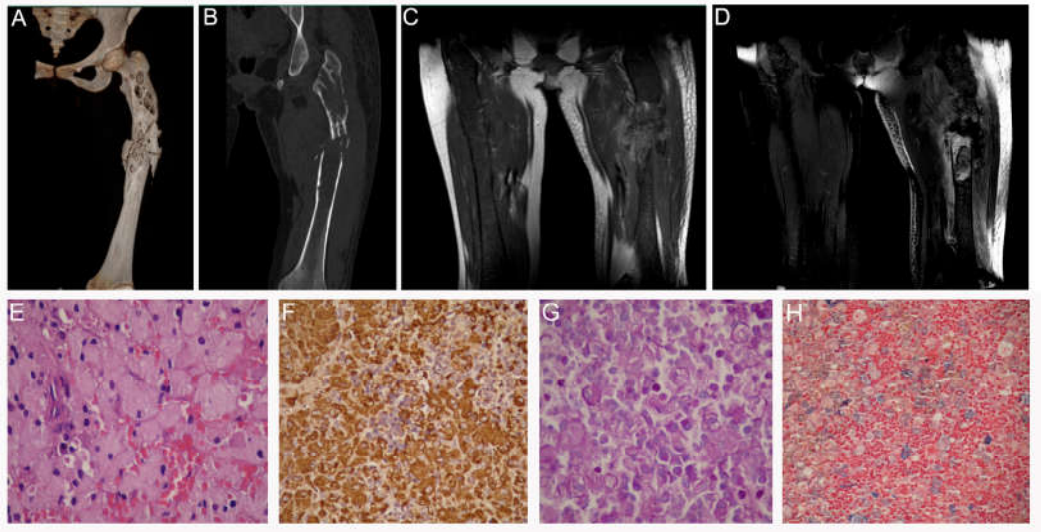

Supplemental Figure S1: Patient 2: (A, B) Initial CT examination revealed dereased intensity of the involved medullary canal and pathological fracture of left femur. (C, D) Subsequent coronal T1 WI showed areas of low SI within the medullary cavity of both femora, the corresponding areas show heterogeneous low SI on the coronal short tau inversion recovery (STIR). Postoperative hematoxylin and eosin staining of the patient revealed Gaucher’s disease (E). Immunohistochemical test of the tumor tissue disclosed positive Kp-1(F), periodic acid－Schiff (G) and iron staining(H).

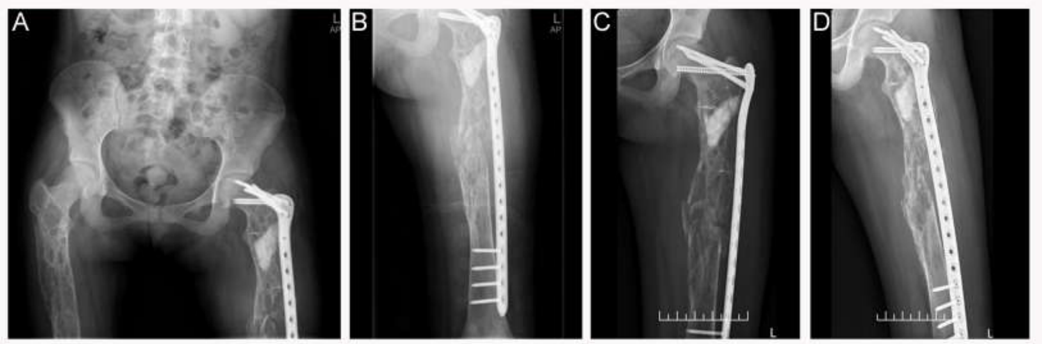

Supplemental Figure S2: (A, B) Postoperative radiograph of the pelvis and femora, demonstrating decreased intensity and disformation of both femora. (C, D) Plane radiograph taken on follow-up (one month after the surgery) indicated the disunion of the fractured femur.

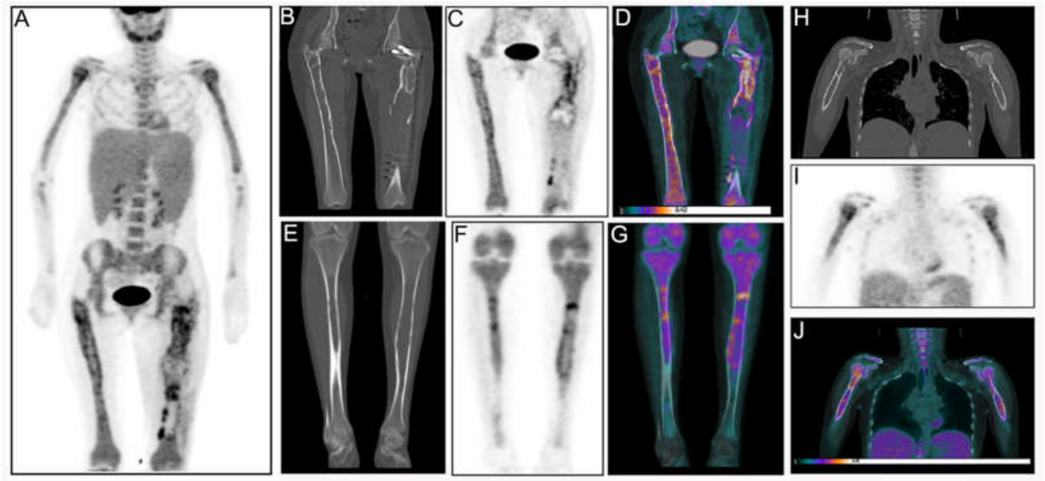

Supplemental Figure S3: Patient 2: (A) Postoperative systematic assessment of disease burden using ^{18}F-FDG PET/CT. Maximum intensity projection demonstrated diffuse uptake of tracer in the extremities, axial skeletons, iliac bones, liver and spleen. Hepatosplenomegaly was also observed. (B-G) CT, PET and fusion images of both femora and tibiae. (H-J) CT, PET and fusion images of bilateral humeral bones.

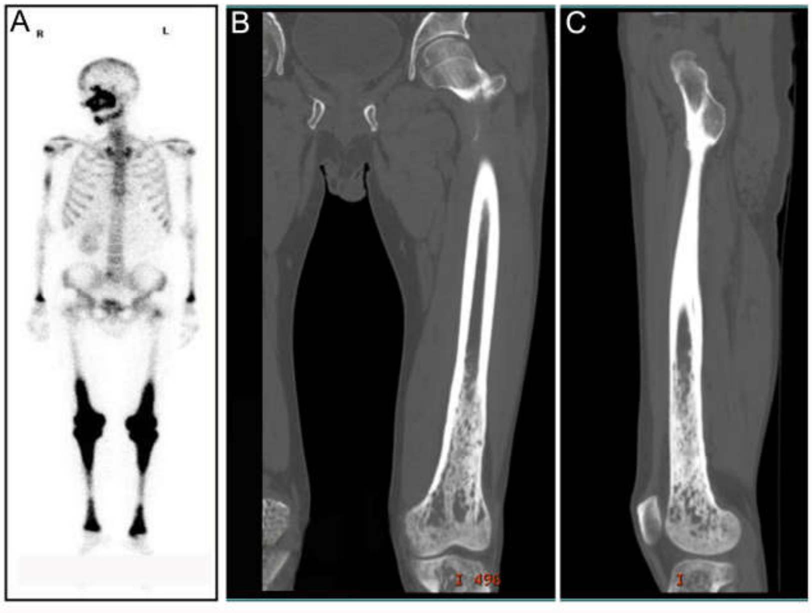

Supplemental Figure S4: Patient 5: (A) ^{99m}Tc-MDP bone scan demonstrated symmetric uptake of radiotracer in the distal femora, tibial metaphyses. (B, C) Coronal and sagittal CT showed osteosclerosis and narrowed marrow cavity in the distal femur.

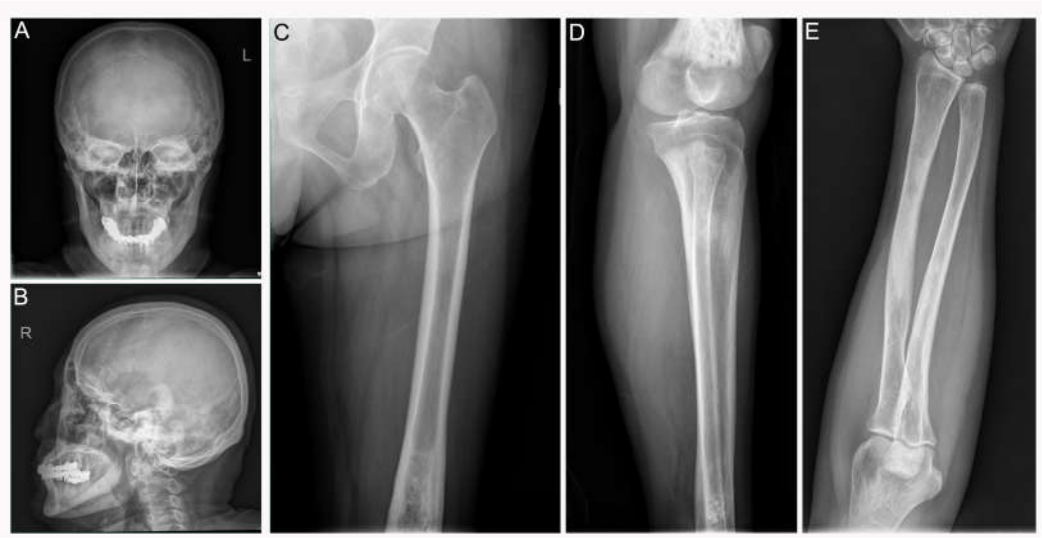

Supplemental Figure S5: Patient 7: Anteroposterior position (A) and lateral position (B) plain film of the skull. Femora and tibiae of the patient were affected. Diaphyseal osteosclerosis of the distal femur (C), proximal tibia (D), ulna and radius (E) as well as narrowing of the correponding marrow cavities were observed on X-ray plain film.

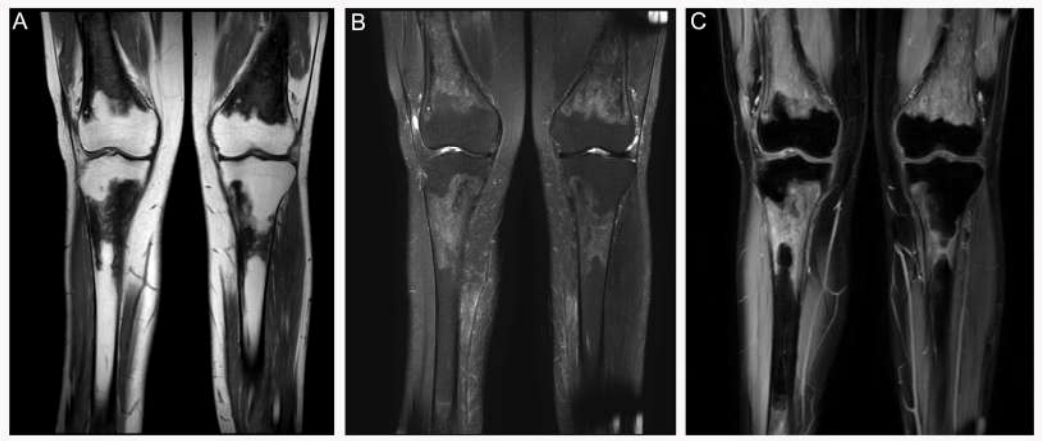

Supplemental Figure S6: Patient 7: MRI imaging of the lower extremities indicated that bilateral patchy diseases appeared as low SI on T1 WI (A) and heterogeneous high SI on T2 WI (B). The MRI documented a homogeneous intense enhancement of the patchy diseases after gadolinium administration (C).

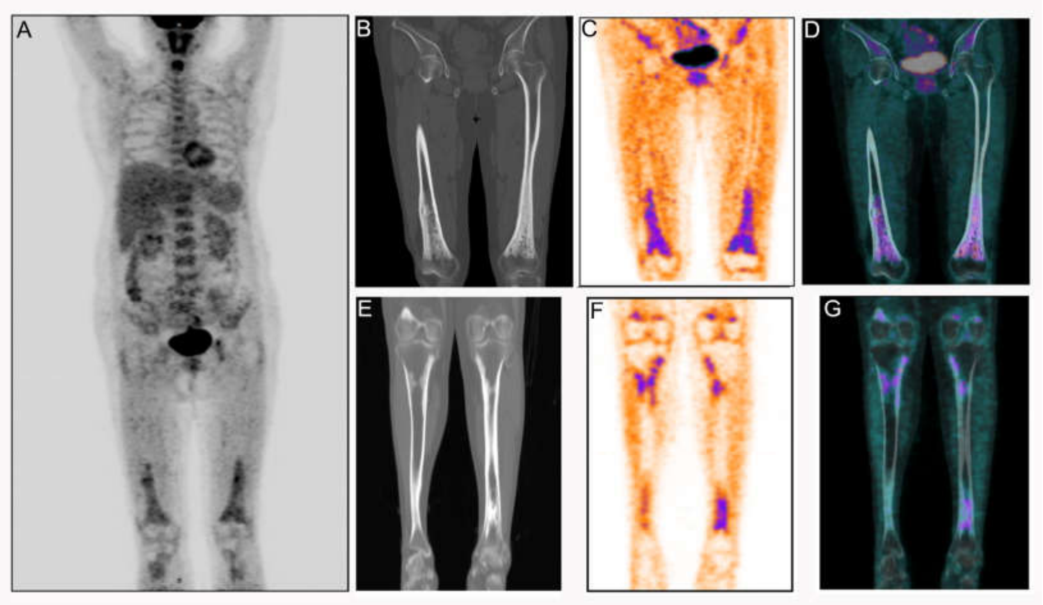

Supplemental Figure S7: Patient 7: (A) ^{18}F-FDG-PET/CT demonstrated high glucose avidity of the metaphyses of lower extremities with a SUVmax of 2.4. (B-D) Cortical substance of bilateral distal femora thickened and corresponding marrow cavities narrowed accordingly, accompanied by increased uptake of radiotracer. (E-G) Similar changes regarding bone formation and glocuse metabolism were observed in both tibiae.
